# Optical Manipulation of Incident Light for Enhanced Photon Absorption in Ultrathin Organic Photovoltaics

**DOI:** 10.3390/nano12223996

**Published:** 2022-11-13

**Authors:** Seungyeon Han, Hyunsung Jung, Hyeon Jin Jung, Bu Kyeong Hwang, In Pyo Park, Su Zi Kim, Dea-Hee Yun, Seog-Young Yoon, Soo Won Heo

**Affiliations:** 1 Nano Convergence Materials Center, Emerging Materials R&D Division, Korea Institute of Ceramic Engineering and Technology (KICET), 101 Soho-ro, Jinju-si 52851, Gyeongsangnam-do, Korea; 2 Department of Materials Science and Engineering, Pusan National University, Busan 46241, Korea; 3 Resetcompany Co., Ltd., Dallaenae-ro, Sujeong-gu, Seongnam-si 13449, Gyeonggi-do, Korea

**Keywords:** organic photovoltaics, ultrathin substrate, CYTOP, optical manipulation, soft nanoimprinting lithography, propagating surface plasmon–polariton, transverse magnetic

## Abstract

We attempted to improve the photon absorption of the photoactive layer in organic photovoltaic (OPV) devices by device engineering without changing their thickness. Soft nanoimprinting lithography was used to introduce a 1D grating pattern into the photoactive layer. The increase in photocurrent caused by the propagating surface plasmon–polariton mode was quantitatively analyzed by measuring the external quantum efficiency in transverse magnetic and transverse electric modes. In addition, the introduction of an ultrathin substrate with a refractive index of 1.34 improved photon absorption by overcoming the mismatched optical impedance at the air/substrate interface. As a result, the power conversion efficiency (PCE) of an ultrathin OPV with a 400 nm grating period was 8.34%, which was 11.6% higher than that of an unpatterned ultrathin OPV, and the PCE was 3.2 times higher at a low incident light angle of 80°, indicating very low incident light angle dependence.

## 1. Introduction

Ultrathin organic photovoltaics (OPVs) with a thickness of less than 10 μm are easy to manufacture with a large area; they have excellent mechanical flexibility and superior power-per-weight characteristics, as well as a free form factor compared to OPVs witha rigid substrate [1,2,3,4]. Therefore, they can be applied as a power source for wearable devices such as portable devices, soft robotics, and human-body-attached biosensors, making them a promising candidate to replace existing energy converters [1,5,6]. However, because of the nature of OPVs based on organic materials, the power conversion efficiency (PCE) tends to depend on the thickness of the photoactive layer. As the thickness of the photoactive layer increases, the photon absorption efficiency increases, but the charge recombination loss also increases because of the low carrier mobility of active materials [7,8]. In contrary, as the photoactive layer becomes thinner, the charge extraction improves, and charge recombination decreases, but the photon absorption decreases [9].

Therefore, to reduce the effects of the photoactive layer thickness on the trade-off between photon absorption and charge extraction, it is necessary to introduce a device structure for the effective optical manipulation of incident light. For example, introducing an antireflection layer reduces the reflected light intensity by addressing the mismatched optical impedance at the air/substrate interface, which increases the photon absorption by the photoactive layer [10,11]. Front and back electrodes for the effective transmission and trapping of incident light are also being studied. By introducing nanostructures into an indium tin oxide or indium zinc oxide transparent electrode, the light trapping effect and electrical properties can be simultaneously improved [12,13]. Furthermore, localized surface plasmon resonance can be obtained by introducing metal nanoparticles into the interlayer and/or photoactive layer [14,15,16,17,18,19,20,21,22], and the propagating surface plasmon–polariton (SPP) effect occurs at the interface between the photoactive layer and metal electrode; these effects can greatly improve the absorption performance [1,9,23].

In particular, when an SPP is excited at the surface of a momentum-matched metal grating by transverse magnetic (TM) polarization, it propagates laterally along the OPV layer, effectively increasing the OPV layer thickness and thus increasing absorption [24]. Because the physical thickness does not change, the carrier dynamics are not affected. Therefore, we introduced an amorphous fluoropolymer (CYTOP) with a refractive index of 1.34 as an ultrathin substrate to efficiently utilize incident light. Furthermore, a 1D grating pattern was applied to the photoactive layer to obtain the propagating SPP effect at the Ag electrode interface, which improves the photon absorption without increasing the layer thickness. Furthermore, by introducing 1D grating patterns with periods of 760, 600, 500, and 400 nm (duty cycle: 0.5) through soft nanoimprinting lithography using a polydimethylsiloxane (PDMS) stamp, the variation in the electrical and optical properties with the period was analyzed. In addition, the absorption, external quantum efficiency (EQE), and PCE in TM and transverse electric (TE) modes were analyzed using polarized light. As a result, we were able to quantitatively analyze the effect of TM and TE according to the pattern period for the first time, indicating that the absorption property can be controlled in a very simple process. The PCE of an ultrathin OPV with a 1D grating pattern that has a period of 400 nm was 8.34%, which was 11.6% higher than that of an unpatterned ultrathin OPV. In addition, the PCE was 3.2 times higher under light with a low incident angle of 80°, indicating very low incident light angle dependence.

## 2. Results and Discussion

**Figure 1:**
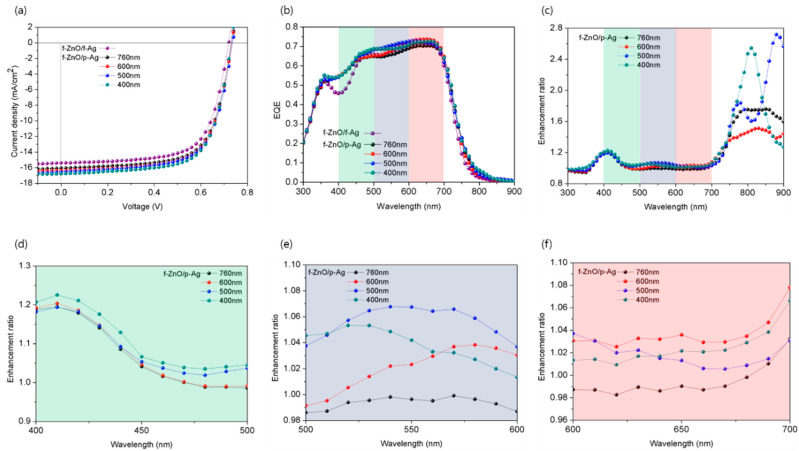
Characteristics of unpatterned ultrathin organic photovoltaic (OPV) and OPVs with various pattern periods. (**a**) *J*-*V* curves under AM 1.5 G illumination at 100 mW/cm^2^, (**b**) external quantum efficiency (EQE) spectra, (**c**) enhancement ratio of EQE, and (**d**–**f**) enlarged graph of the colored part of the enhancement ratio of EQE data in (**c**).

Figure 1 shows the *J*–*V* characteristics, EQE, and enhancement ratio of the EQE of ultrathin OPVs with various 1D grating periods and devices without nanostructures. Enlarged views of some of these data are also shown. The detailed device fabricating process is shown in Figure S1. Figure S2 shows the Si mold (template) that was fabricated to add 1D grating patterns to the photoactive layer, the PDMS stamp that was fabricated using the mold, and the photoactive layer to which the patterns were applied. In addition, the structure of the patterned photoactive layer and the surface morphology measured by atomic force microscopy (AFM) are shown in Figure S3. Table 1 presents the short-circuit current density (*J*_SC_), open-circuit voltage (*V*_OC_), fill factor (FF), and PCE of the fabricated devices. The determination method for series resistance (*R*_s_) and shunt resistance (*R*_sh_) presented in the table is shown in Figure S4. Data are displayed with the average and deviation of the values of 10 or more cells (Figure S5). The *J*_SC_ values calculated from both the *J*–*V* curve and the EQE are presented; the discrepancy between them did not exceed 4%. In addition, the *J*_SC_ values calculated from the EQEs measured in TM and TE modes are given. The *J*_SC_, *V*_OC_, and FF of the ultrathin OPV without a 1D grating pattern (f-ZnO/f-Ag, reference) were 15.26 mA/cm^2^, 0.72 V, and 67.9%, respectively. The calculated PCE was 7.47%. For the ultrathin OPV (f-ZnO/p-Ag) with a 1D grating pattern, as the pattern period decreased, *J*_SC_ increased to 16.06, 16.23, 16.55, and 16.78 mA/cm^2^ for pattern periods of 760, 600, 500, and 400 nm, respectively. The maximum PCE of 8.34% was obtained at a period of 400 nm. *V*_OC_ and the FF exhibited negligible changes when nanostructure was introduced. These results confirm that the introduction of the 1D grating pattern increased *J*_SC_ and thus improved the PCE. To determine the reason for the increase in *J*_SC_, we measured the EQE (Figure 1b). Interestingly, the wavelength region in which the EQE was enhanced depended on the period of the 1D grating pattern. Specifically, patterns with periods of 600, 500, and 400 nm showed increased EQE mainly in the 600–700 nm, 500–600 nm, and 400–500 nm regions, respectively (Figure 1c–f). However, the OPV with a period of 760 nm showed a smaller increase compared to the other nanostructures.

**Figure 2:**
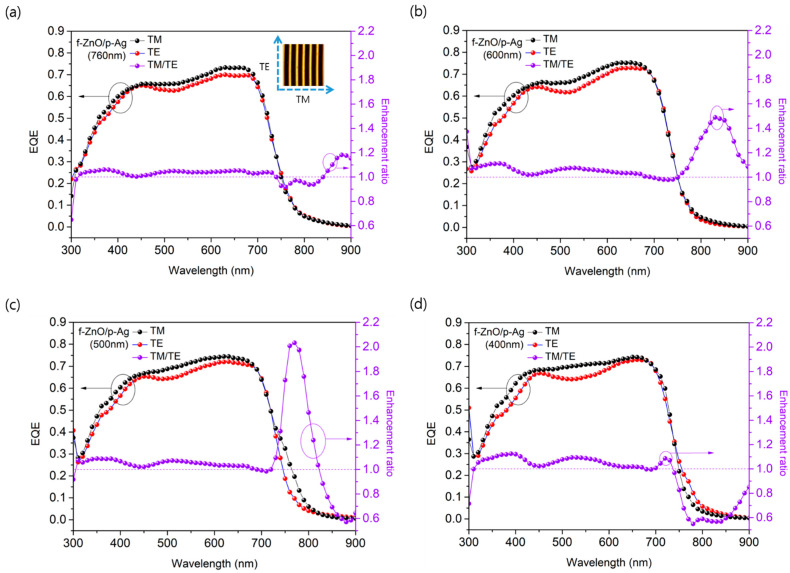
EQE spectra and EQE enhancement ratio of ultrathin OPVs measured in transvers magnetic (TM) and transvers electric (TE) modes for pattern period of (**a**) 760 nm, (**b**) 600 nm, (**c**) 500 nm, and (**d**) 400 nm. Enhancement ratio was calculated by dividing the TM value by the TE value. The inset in (**a**) illustrates the TM and TE modes.

We measured the EQE in TM and TE modes to determine why the 1D grating period affected *J*_SC_, and the difference between the two values was calculated as the enhancement ratio (Figure 2). The measurement method used in TM and TE modes is illustrated in Figure S6. The TE portion, which occupies half of sunlight, causes weak SPP effect [16,25]. Therefore, the EQE measured using unpolarized light should be similar to the average of the EQEs measured in TM and TE modes. Consequently, the SPP effect can be expected to be larger when the difference between the *J*_SC_ values calculated for the TM and TE modes is larger. Thus, it is possible to quantitatively analyze the effect of the period of the 1D grating pattern on the SPP effect. For a pattern period of 760 nm (Figure 2a), the difference between the *J*_SC_ values for the TM and TE modes was 0.53 mA/cm^2^, which was not significant. The average of the *J*_SC_ values calculated for both modes was also very similar to the *J*_SC_ value obtained from the EQE measured using unpolarized light; thus, the experimental error was negligible. By contrast, as the 1D grating period decreased, the difference between TM and TE modes increased to 0.7, 0.86, and 0.99 mA/cm^2^ for periods of 600, 500, and 400 nm, respectively. Therefore, we can conclude that the increase in *J*_SC_ is caused by the increase in the SPP effect due to a decrease in the 1D grating period.

**Figure 3:**
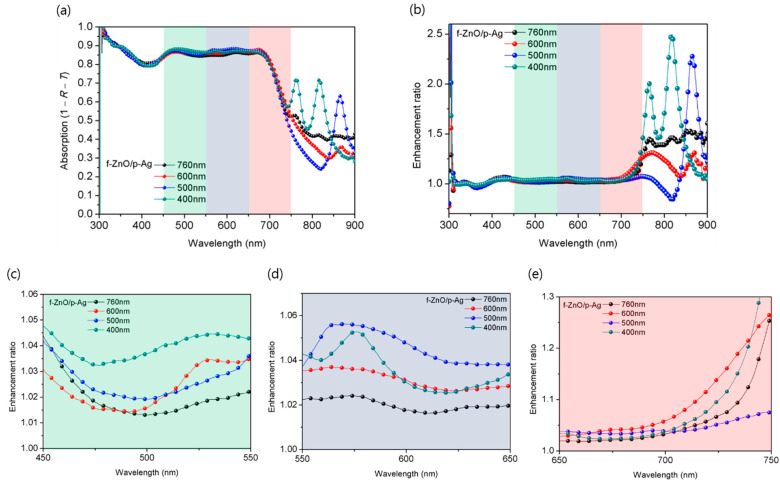
(**a**) Absorption spectra and (**b**) absorption enhancement ratio of ultrathin OPVs with pattern periods of 760, 600, 500, and 400 nm. Enhancement ratio was calculated by dividing the values of each patterned OPV by the value of the reference device. (**c**–**e**) Enlarged view of the colored part of enhancement ratio in (**b**).

In addition, we measured and calculated the reflectance (*R*) and transmittance (*T*) of the devices to observe the absorption in the photoactive layer according to the period of the 1D grating pattern. The results and the enhancement ratio are shown in Figure 3. As the pattern period decreased, the area of the enhancement ratio was blue-shifted (Figure 3c–e), which is similar to the behavior of the EQE. Figure 3a,b shows that the strong increase in absorption above 750 nm results from the SPP effect caused by the nanostructure on the photoactive layer, which increases the absorption area of the PTB7: PC_71_BM system. Therefore, the introduction of the 1D grating pattern on the photoactive layer enables the SPP effect in TM mode and extends the absorption region. Consequently, the photon absorption could be increased without increasing the thickness of the photoactive layer. This result shows that the absorption region can be modulated simply by varying the pattern period, and there is no need to adjust the absorption region by band gap tuning through molecular tailoring of the photoactive material. In addition, as the pattern period decreased, the contact area between the photoactive layer and metal electrode increased, reducing charge recombination at the interface, and the *R*_sh_ increased from 6.3 kΩ cm^2^ (reference device) to 8.6 kΩ cm^2^ (device with a grating period of 400 nm). Thus, the internal resistance of the device decreased, resulting in a decrease in the *R*_s_, which decreased by 3.08 Ω cm^2^ in the device with a pattern period of 400 nm [26]. As a result, *J*_SC_ increased by 10%, from 15.26 to 16.78 mA/cm^2^, after the pattern was applied.

**Figure 4:**
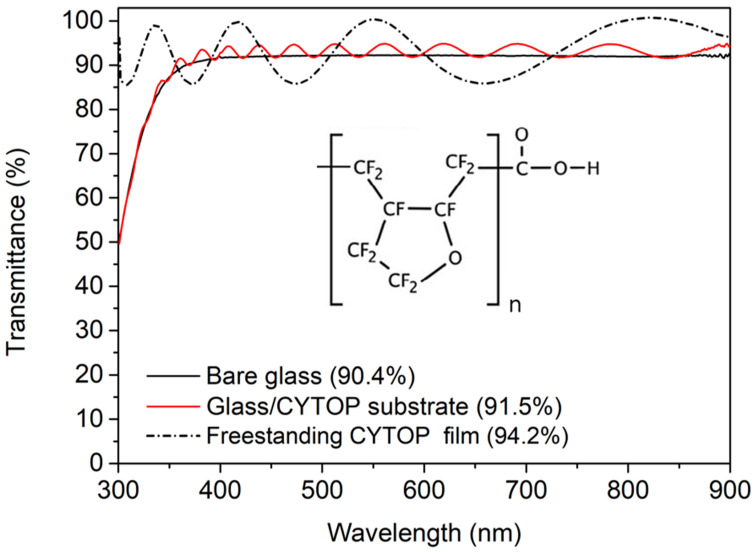
Transmittance spectra of bare glass, a glass/amorphous fluoropolymer (CYTOP) substrate, and a freestanding CYTOP film. Inset shows the chemical structure of CYTOP.

In addition, the amount of photons incident on the photoactive layer was increased by refractive index engineering. CYTOP has a lower refractive index (1.34) than conventional materials such as glass (~1.5), polycarbonate (1.58), poly(ethylene terephthalate) (1.66), and poly(ethylene2, 6-naphthalene dicarboxylate) (1.75). When CYTOP is applied to an ultrathin substrate, the reflection loss at the air/substrate interface can be reduced [27]. As shown in Figure 4, the transmittance of bare glass in the visible region is 90.4%. However, the refractive index gradually increases when a CYTOP coating is added. CYTOP acts as an antireflection layer, increasing the transmittance. In addition, when the glass substrate is removed, the transmittance of the CYTOP substrate increases to 94.2%, and the photon absorption is greatly improved without an increase in the thickness of the photoactive layer. Therefore, the PCE of an unpatterned ultrathin OPV to which CYTOP was applied is 4.4% higher than that of an unpatterned OPV using rigid glass (7.15%) (not included in the table; *J*_SC_: 14.6 mA/cm^2^, *V*_OC_: 0.72 V, FF: 67.8%). This difference is similar to the 4.5% difference in photocurrent between the two devices. The increase in photocurrent observed in the device after CYTOP application is thus attributed to the increased substrate transmittance. In addition, the device shows high transmittance even at 350 nm or less; consequently, it can be used as a substrate material in UV-sensitive sensors, photodiodes, phototransistors, etc.

**Figure 5:**
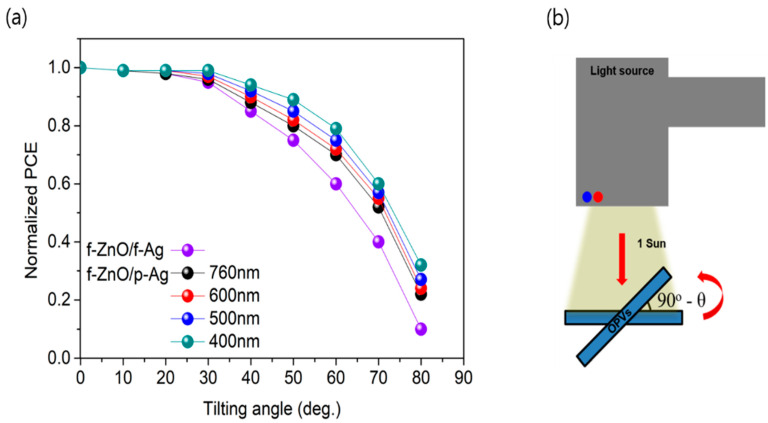
(**a**) Incident angle dependence of reference device and OPVs with various pattern periods. Data show normalized PCEs. (**b**) Setup for incident angle dependence measurement. A tilt angle of 0° means that the light is incident vertically.

Figure 5 shows the measured incident angle dependence of the reference device and OPVs with various pattern periods. When the tilt angle of the incident light was approximately 20°, the normalized PCEs with and without nanostructure did not differ significantly. However, the difference became apparent as the tilt angle increased. In particular, at a high tilt angle of 80°, the normalized PCE of the unpatterned ultrathin OPV was 0.1, whereas that of the device with a grating period of 400 nm was 0.32, which is 3.2 times larger. As the period of the 1D grating pattern decreases, the dependence on the incident angle of light decreases because the presence of a denser nanopattern is more effective for the scattering of incident light, resulting in a longer light path and more efficient exciton generation. Therefore, the increase in photocurrent is thought to directly cause the improvement in the PCE.

## 3. Materials and Methods

### 3.1. Materials

Zinc acetate dihydrate, 2-methoxymethanol, monoethanolamine (MEA), chlorobenzene (CB), and 1,8-diiodooctane (DIO) were purchased from Merck (Darmstadt, Germany). PTB7 as a donor polymer and PC_71_BM (>99%) as an acceptor material were purchased from 1-Material (Dorval, QC, Canada) and Solenne BV (Groningen, Netherlands), respectively, and used as received. CYTOP was purchased from AGC (CTX-809SP2, Tokyo, Japan) and used after filtering with a 5 μm syringe filter. UV-curable PDMS (Shin-Etsu, X-34-4184-A/B, Tokyo, Japan) was used as received. Ag and MoO_3_ for electrodes were purchased from iTASCO (Seoul, Korea).

### 3.2. Synthesis of ZnO Solution

The ZnO solution was synthesized using a previously reported method [9]. Briefly, zinc acetate dihydrate (99.999%, 1 g) and MEA (Merck, >99.9%, 0.28 g) were dissolved in 10 mL of 2-methoxyethanol (99.8%) at 65 °C for 12 h. A 0.2 μm syringe filter was used to filter the ZnO solution.

### 3.3. Fabrication of PDMS Stamps with 1D Grating Patterns

To fabricate PDMS stamps with various 1D grating patterns, we fabricated Si templates with periods of 760, 600, 500, and 400 nm. The duty cycle of the 1D grating pattern was 0.5, and the Si template was fabricated by electron beam patterning (Vistec EBPG 5000ES system, Eulitha, Switzerland). First, a UV-curable PDMS mixture (X-34-4184-A/B, mixing ratio 1:1) was degassed in a vacuum chamber for 10 min. Then, the degassed PDMS mixture was poured onto the Si template, and the sample was degassed again for 10 min. After the sample was irradiated with UV light (365 nm) for 3 h, the PDMS stamp with a 1D grating pattern was peeled from the surface of the Si template.

### 3.4. Device Fabrication

To fabricate the ultrathin substrate, 300 μL of CYTOP was poured onto the cleaned rigid substrate (supporting substrate) and spin-coated at 2000 rpm for 20 s. Because the CYTOP used in this study has an end group substituted with a perfluoro group, it does not require the formation of a self-assembled monolayer for the delamination of ultrathin OPVs, in contrast to the CYTOP used in previous studies. The substrates were soft-baked on a hotplate at 80 °C and thermally annealed at 150 °C for 30 min to remove all residual solvent. The thickness of the fabricated ultrathin substrate was 1 μm. The hydrophilicity of the prepared ultrathin substrate was modified by UV ozone cleaning. Indium tin oxide was deposited to a thickness of 100 nm by sputtering and was used as a transparent electrode. Ultrathin OPVs with and without 1D grating patterns were fabricated, as shown in Figure S1. In the flat ZnO layer, the ZnO solution was spin-coated on the patterned ITO glass at 2000 rpm for 30 s and thermally annealed on a 150 °C hotplate for 10 min to obtain a thickness of approximately 30 nm. To fabricate the photoactive layer, PTB7 and PC_71_BM were used as the electron donor and electron acceptor, respectively. They were dissolved in 1 mL of CB at a weight ratio of 1:1.5 (10 mg:15 mg), and 3 vol% of DIO was added as a processing additive. The photoactive solution was spin-coated on the ZnO layer at 800 rpm for 7 s in a glove box filled with N_2_. The thickness of the photoactive layer was 100 nm. To form a nanostructure, the patterned PDMS was gently placed on the spin-coated photoactive layer and removed after 60 s. No pressure was applied to the patterned PDMS. The pattern depth of the 1D gratings with various pattern periods was 55 nm, respectively. Furthermore, a metal electrode was formed without thermal annealing of the photoactive layer even after patterning. The metal electrode was obtained by thermal evaporation. A MoO_3_ hole-transporting layer (12 nm) and Ag electrodes (100 nm) were deposited by thermal evaporation (0.1 and 0.5 Å/s, respectively) under a high vacuum (~10^−4^ Pa) using a metal mask.

### 3.5. Measurements

The film thicknesses of the fabricated ZnO and photoactive layers were measured using a surface profiler (DEKTAK 6M, Bruker, Billerica, MA, USA). Transmission and reflection data for the calculation of the absorption in TM and TE modes were measured using a UV–vis spectrophotometer (V-650 and V-670, JASCO, Tokyo, Japan) with an integrating sphere, and a polarizing filter (SPF-50C-32, OptoSigma, Santa Ana, CA, USA) was used for a light source. AFM (5400 scanning probe microscope, Agilent Technologies, Santa Clara, CA, USA) in tapping mode was used to observe the surface morphology of the patterned photoactive layers. The *J*–*V* characteristics of the devices were measured using a Keithley 2400 source measure under simulated solar illumination (AM 1.5, 100 mW/cm^2^) from a solar simulator based on a 1000 W Xe lamp (Oriel, Irvine, CA, USA). The light intensity was calibrated using a standard silicon solar cell (BS520, Bunkoh-Keiki, Tokyo, Japan). The active area of each device was defined using a 0.04 cm^2^ metal photomask. The EQE of each device was measured using a Polaronix K3100 IPCE measurement system (McScience, Seoul, Korea). A light source passed through a polarizing filter was used to measure the EQE in TM and TE modes.

## 4. Conclusions

We improved the properties of ultrathin OPVs by relatively simple device engineering to improve the photon absorption without changing the thickness of the photoactive layer. When a 1D grating pattern was applied to the photoactive layer by soft nanoimprinting lithography, the obtained SPP mode improved the electrical and optical properties. In addition, we demonstrated that the absorption region can be modified using a simple method by controlling the pattern period instead of material engineering methods such as the molecular tailoring of the photoactive materials. Furthermore, there was a slight refractive index mismatch between air and the CYTOP used as the substrate, which increased the amount of photons incident on the photoactive layer, improving the device photocurrent. In addition, as the pattern period decreased, the dependence on the incident angle of light decreased; thus, the devices can be applied in skin-attached sensors with high dynamic activity and power supplies for portable devices.

## Figures and Tables

**Table 1:** Performance of unpatterned ultrathin OPV and OPVs with 1D gratings with different pattern periods measured under AM 1.5 G illumination at 100 mW/cm^2^.

Nanostructure		*J*_SC_ (mA/cm^2^) (Calc. from EQE)	* J_SC_ (mA/cm^2^) (Calc. from EQE in TM and TE Modes)	*V*_OC_ (%)	FF (%)	PCE (%)	** *R*_s_ (Ω cm^2^)	** *R*_sh_ (kΩ cm^2^)
Reference (f-ZnO/f-Ag)		15.26 ± 0.02 (15.04)	TM 15.07	0.72	67.9 ± 0.4	7.47 ± 0.11	3.45	6.3
			TE 15.03					
Pattern period of 1D grating (f-ZnO/pAg)	760 nm	16.06 ± 0.09 (15.56)	TM 15.85	0.73	67.8 ± 0.3	7.95 ± 0.04	3.32	6.9
			TE 15.32					
	600 nm	16.23 ± 0.05 (15.79)	TM 16.16	0.73	68.0 ± 0.3	8.06 ± 0.09	3.23	7.2
			TE 15.46					
	500 nm	16.55 ± 0.05 (15.96)	TM 16.38	0.73	68.0 ± 0.1	8.21 ± 0.03	3.15	7.8
			TE 15.52					
	400 nm	16.78 ± 0.08 (16.12)	TM 16.63	0.73	68.1 ± 0.1	8.34 ± 0.04	3.0	8.6
			TE 15.64					

Average values were obtained from at least 10 cells. * EQE was measured in TM and TE modes using light passed through a polarizing filter. ** *R*_sh_ and *R*_s_ are the slopes of *J*–*V* curves obtained in the dark at 0 and 1 V, respectively.

## Data Availability

The data presented in this investigation is available in article or from the corresponding authors.

## Supplementary Materials

The following supporting information can be downloaded at: https://www.mdpi.com/article/10.3390/nano12223996/s1, Figure S1: Schematic illustration of the fabrication process for ultrathin OPVs with 1D grating pattern by soft imprinting lithography using a PDMS mold with various pattern period. Inset figures present (a) AFM images for 1D grating patterned photoactive layers (period: 760, 600, 500, and 400 nm, depth: 55 nm), chemical structurers (b) PTB7, and (c)PC_71_BM, respectively.; Figure S2: Pictures show the (a) Si mold with various pattern periods of 1D gratings, (b) configuration of 1D grating patterns in Si mold, (c) patterned PDMS stamp, and (d) patterned photoactive layer on the ITO glass/supporting substrate.; Figure S3: (a) Structure of the patterned photoactive layer. AFM images of patterned photoactive layers with various 1D gratings of (b) 760 nm, (c) 600 nm, (d) 500 nm, and (e) 400 nm, respectively. Inset shows the 3D image of various pattern period of patterned photoactive layer.; Figure S4: Determination method for series resistance and shunt resistance.; Figure S5: PCE distributions according to the ultrathin OPVs with (a) non-patterned structure, 1D grating patterns of (b) 760 nm, (c) 600 nm, (d) 500 nm, and (e) 400 nm, respectively.; Figure S6: Schematic diagram of the measurement methods for (a) TM mode, and (b) TE mode.
