# Supplementary material for: Optical Manipulation of Incident Light for Enhanced Photon Absorption in Ultrathin Organic Photovoltaics
Source: Nanomaterials (Basel). 2022 Nov 13;12(22):3996. doi: 10.3390/nano12223996 (PMC9696273; doi:10.3390/nano12223996)
Supplement: Supplementary file 1 [file nanomaterials-12-03996-s001.zip › nanomaterials-2036831-supplementary.pdf]

Supplementary Materials

Optical Manipulation of Incident Light for Enhanced Photon Absorption in Ultrathin Organic Photovoltaics

Seungyeon Han [1,2], Hyunsung Jung [1], Hyeon Jin Jung [1], Bu Kyeong Hwang [1], In Pyo Park [1], Su Zi Kim [1], Dea-Hee Yun [3], Seog-Young Yoon [2,*] and Soo Won Heo [1,*]

[1] Nano Convergence Materials Center, Emerging Materials R&D Division, Korea Institute of Ceramic Engineering and Technology (KICET), 101 Soho-ro, Jinju-si 52851, Gyeongsangnam-do, Korea

[2] Department of Materials Science and Engineering, Pusan National University, Busan 46241, Korea

[3] Resetcompany Co., Ltd., Dallaenae-ro, Sujeong-gu, Seongnam-si 13449, Gyeonggi-do , Korea

* Correspondence: syy3@pusan.ac.kr (S.-Y.Y.); soowon.heo@kicet.re.kr (S.W.H.)

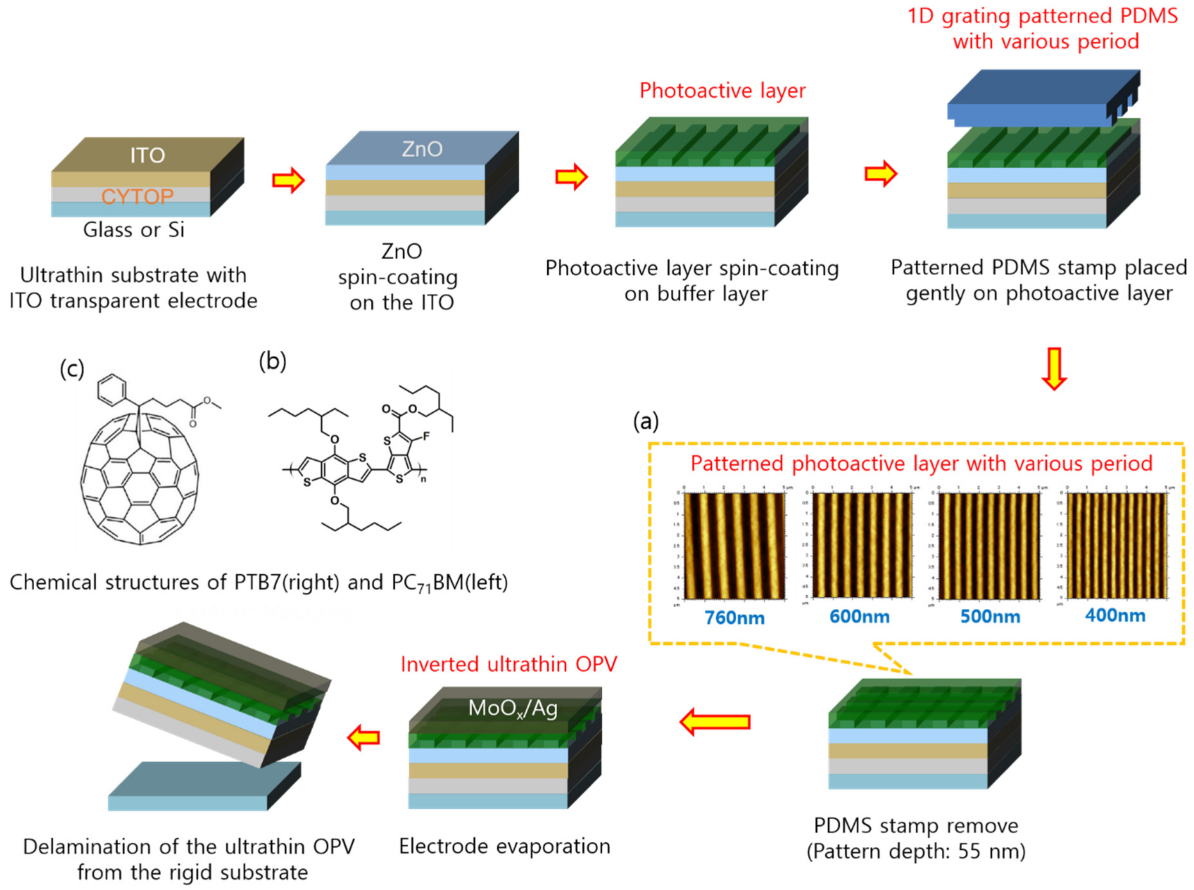

Figure S1. Schematic illustration of the fabrication process for ultrathin OPVs with 1D grating pattern by soft imprinting lithography using a PDMS mold with various pattern period. Inset figures present (a) AFM images for 1D grating patterned photoactive layers (period: 760, 600, 500, and 400 nm, depth: 55 nm), chemical structurers (b) PTB7, and (c)$PC_{71}BM$, respectively.

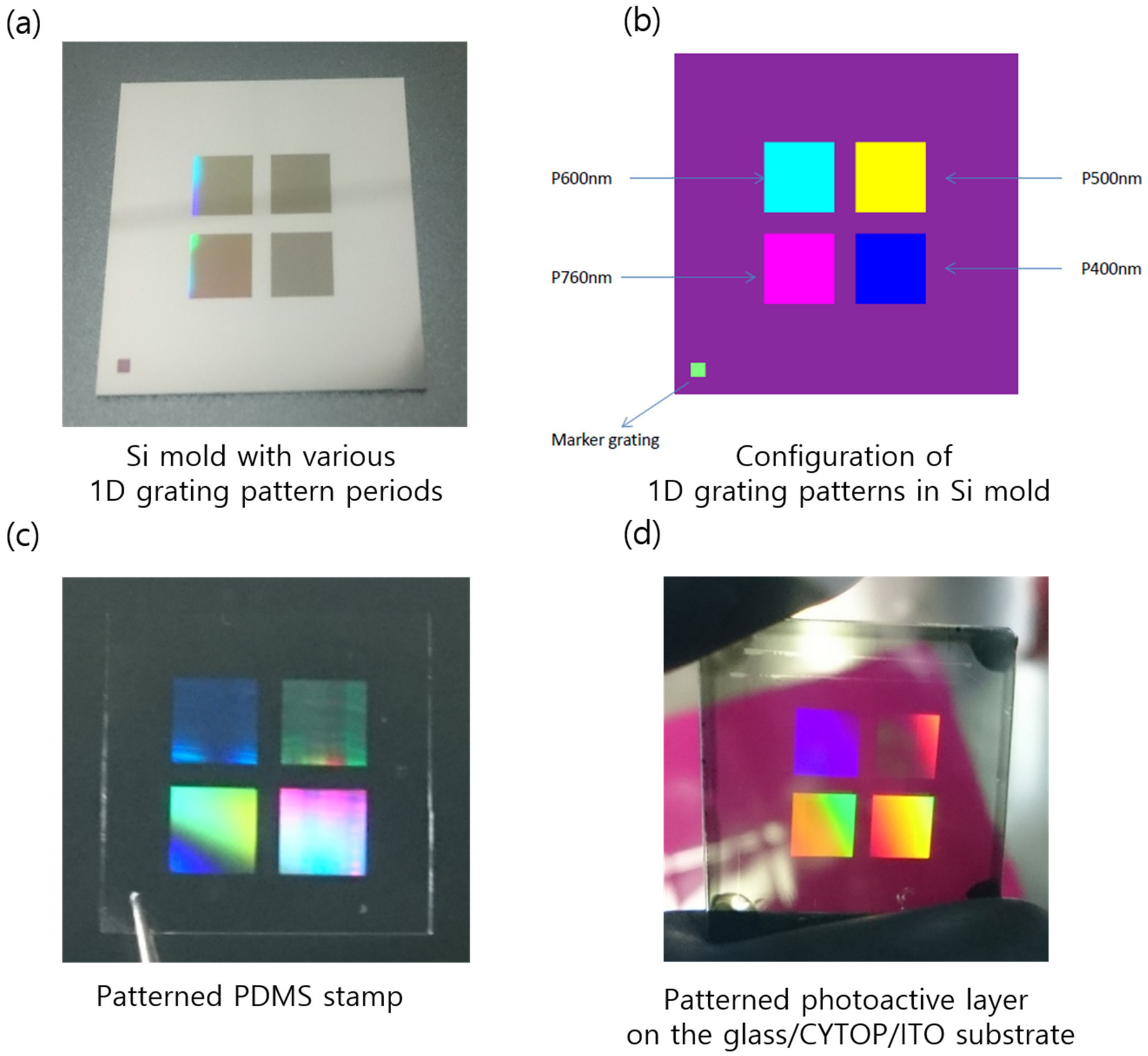

Figure S2. Pictures show the (a) Si mold with various pattern periods of 1D gratings, (b) configuration of 1D grating patterns in Si mold, (c) patterned PDMS stamp, and (d) patterned photoactive layer on the ITO glass/supporting substrate.

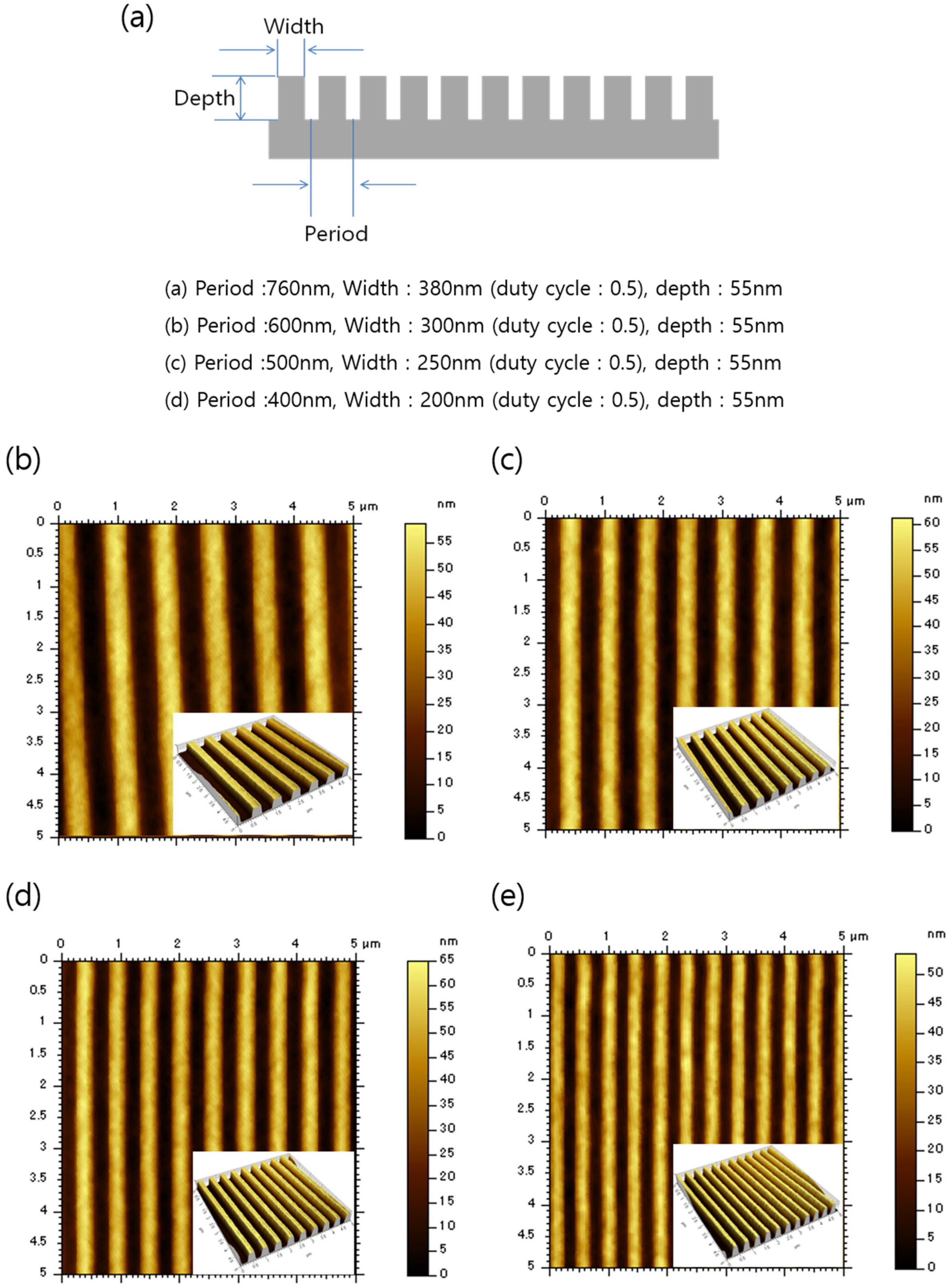

Figure S3. (a) Structure of the patterned photoactive layer. AFM images of patterned photoactive layers with various 1D gratings of (b) 760 nm, (c) 600 nm, (d) 500 nm, and (e) 400 nm, respectively. Inset shows the 3D image of various pattern period of patterned photoactive layer.

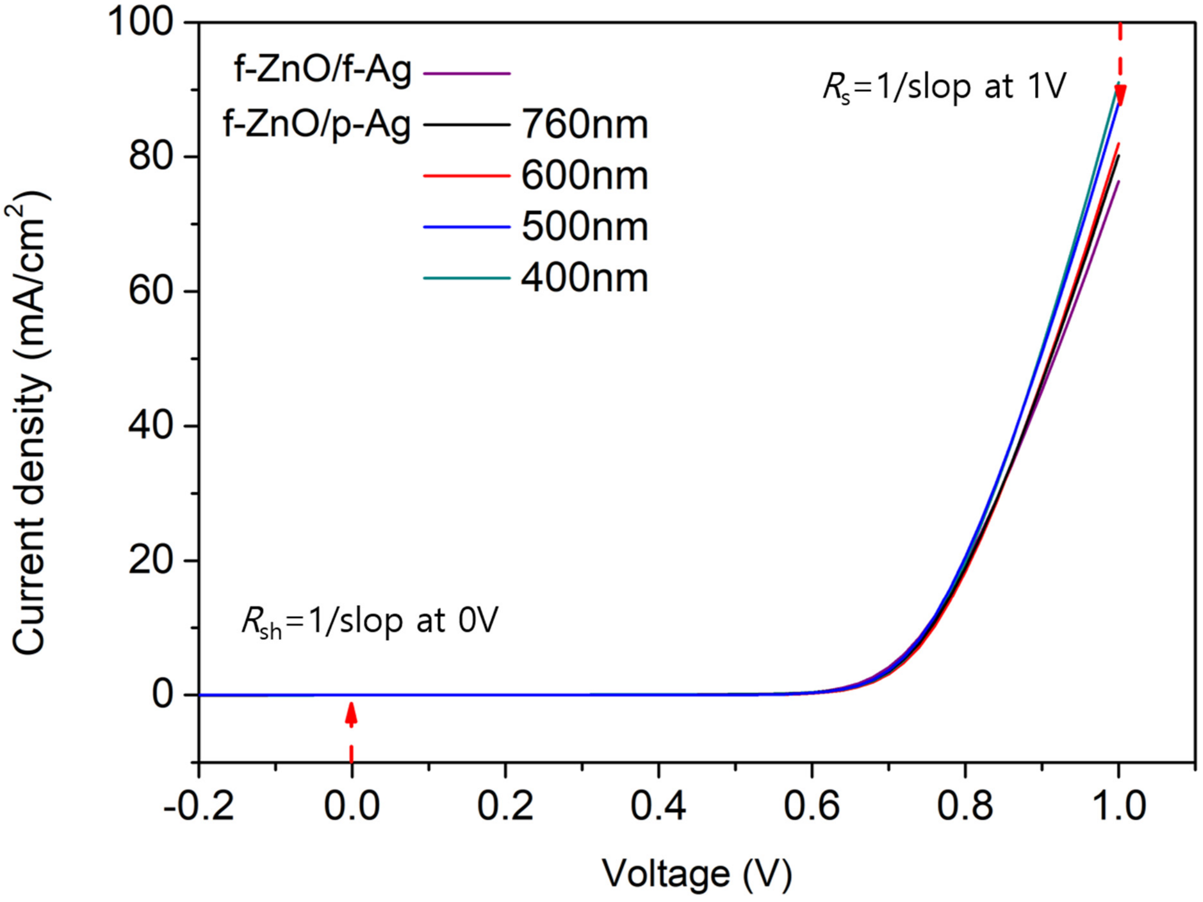

Figure S4. Determination method for series resistance and shunt resistance.

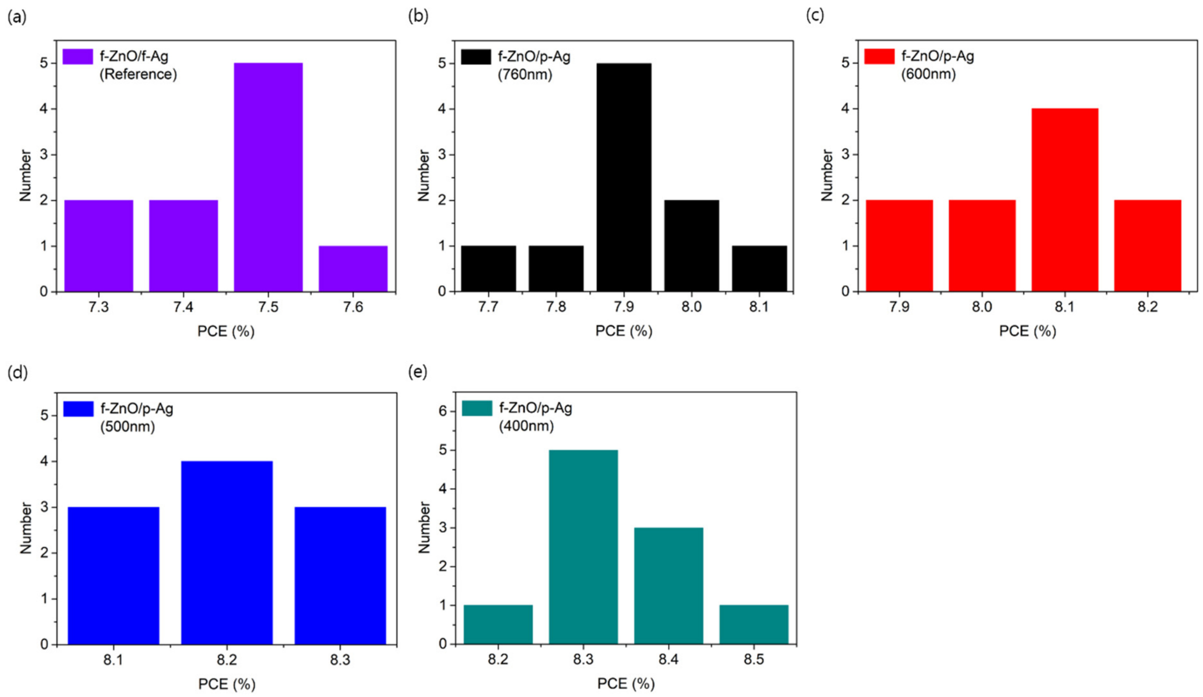

Figure S5. PCE distributions according to the ultrathin OPVs with (a) non-patterned structure, 1D grating patterns of (b) 760 nm, (c) 600 nm, (d) 500 nm, and (e) 400 nm, respectively.

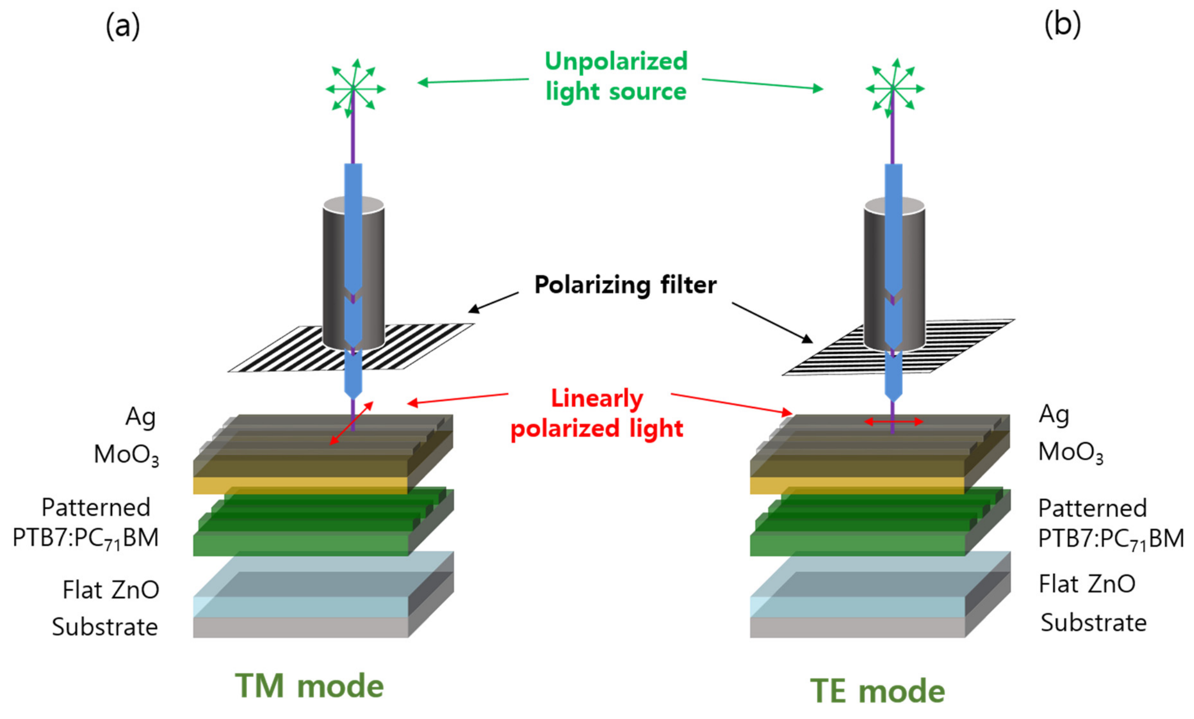

Figure S6. Schematic diagram of the measurement methods for (a) TM mode, and (b) TE mode.
